# From Information to Vaccination: A Conceptual Framework for Understanding Community Participation in Catch-Up Vaccination for Children in Abidjan, Côte d’Ivoire

**DOI:** 10.3390/vaccines14090786

**Published:** 2026-09-08

**Authors:** Abdoul Gadiry Fadiga, ASM Shahabuddin, Kevin Sylvestre Yohou, Françoise Soro, Mathilde Manouan, Bognan Valentin Kone, Tania N’zi-Boa, Josette Dedoh, Akissi Régine Attia-Konan, Julie Sackou-Kouakou

**Affiliations:** 1 United Nations Children’s Fund (UNICEF), Abidjan 04 BP 443, Côte d’Ivoire; jdedoh@unicef.org; 2 Maternal, Newborn, Child and Adolescent Health Section, Global Health Practice, Child Survival Centre of Excellence, United Nations Children’s Fund, Nairobi P.O. Box 44145–00100, Kenya; ashahabuddin@unicef.org; 3 National Institute of Public Health, Abidjan 01 BP V47, Côte d’Ivoire; yoskev@gmail.com (K.S.Y.); sorofrancoise@yahoo.fr (F.S.); manounatto3@gmail.com (M.M.); konebognan@gmail.com (B.V.K.); tnzi@yahoo.fr (T.N.-B.); attiaregci@gmail.com (A.R.A.-K.); juliekouakou77@gmail.com (J.S.-K.); 4 Faculty of Pharmaceutical and Biological Sciences, Félix Houphouët-Boigny University, Abidjan 01 BP V34, Côte d’Ivoire

**Keywords:** catch-up vaccination, community participation, zero-dose children, vaccination, trust, Côte d’Ivoire

## Abstract

**Background/Objectives**: Catch-up vaccination is essential for reaching zero-dose and under-vaccinated children. However, community participation depends on several factors whose interactions remain insufficiently understood. This study aimed to analyze the dimensions structuring community participation in catch-up vaccination and to develop a conceptual framework describing their relationships. **Methods**: An analytical qualitative study was conducted between July and November 2025 in the health districts of Abobo and Yopougon, Abidjan, Côte d’Ivoire. Data were gathered from 106 participants through 26 semi-structured individual interviews and eight focus group discussions involving 80 parents or caregivers of children aged 0–59 months. Interviews and focus group discussions were transcribed in French, and thematic analysis was conducted using NVivo 14 (Lumivero, Denver, CO, USA). A standardized thematic codebook with operational definitions was used by three coders to ensure consistent coding across transcripts. **Results**: Thematic analysis across participant groups identified seven components shaping community participation: exposure to information, understanding of the message, trust in actors and services, social acceptance, parents’ availability and capacity, participation in the activity, and effective vaccination of the child. Five cross-cutting factors influenced progression along this pathway. Participation could break down when information was insufficient or unclear, trust was undermined by perceived costs or rumors, social acceptance was constrained, or parents faced competing work and time demands. These interactions informed the Community Participation Pathway for Catch-up Vaccination (CPP-CV), which identifies potential disruption points between information exposure and effective vaccination and highlights that non-participation may result from the interaction of informational, social, individual, structural, and organizational factors rather than vaccine hesitancy alone. **Conclusions**: The findings suggest that participation in catch-up vaccination goes beyond information alone and results from the interaction between community and organizational factors. Non-participation should not be treated as a single problem or assumed to reflect vaccine hesitancy. The CPP-CV provides a programmatically oriented framework for identifying where the pathway from information to vaccination breaks down and for tailoring catch-up strategies accordingly.

## 1. Introduction

Child vaccination is one of the most effective public health interventions for reducing morbidity and mortality related to vaccine-preventable diseases. Despite the progress achieved over recent decades, millions of children remain unvaccinated or under-vaccinated, particularly in low- and middle-income countries [1,2]. The Immunization Agenda 2030 (IA2030) has therefore placed equity at the center of global priorities, with the ambition of leaving no child behind and paying particular attention to “zero-dose” children, operationally defined as children who have not received a first dose of a vaccine containing the diphtheria–tetanus–pertussis antigen [1,3].

The disruptions to vaccination services observed during the COVID-19 pandemic widened coverage gaps and increased the number of zero-dose and under-vaccinated children in many countries [2,4]. In response, The Big Catch-Up initiative, launched in 2023 by the World Health Organization (WHO), UNICEF, Gavi, and other partners, aims to reach children who missed vaccinations, restore coverage levels, and strengthen routine immunization programmes [4]. However, the success of such strategies does not depend solely on vaccine availability or on the capacity of services to organize sessions. It also requires that families be informed, accept vaccination, and be able to effectively access the services offered.

In Côte d’Ivoire, gaps in childhood immunization coverage remain a concern. According to the 2021 Côte d’Ivoire Demographic and Health Survey (DHS), 8.6% of children were zero-dose nationally, with a higher proportion in urban areas (10.3%) than in rural areas (6.9%) [5]. This urban disparity is particularly relevant to Abidjan, where densely populated and peri-urban communities may face specific barriers to accessing and completing routine immunization. These persistent gaps reinforce the importance of catch-up vaccination strategies to identify and reach children missed by routine immunization services.

Community participation is therefore an essential dimension of catch-up vaccination strategies. Studies on vaccination have identified many determinants of service use, including access to information, trust in vaccines and institutions, rumors, social and religious beliefs, the influence of relatives and peers, economic constraints, distance, and service organization [6,7,8,9]. Recent studies from high zero-dose settings in sub-Saharan Africa further demonstrate that non-vaccination reflects an interplay of demand- and supply-side factors [10,11]. These include inadequate information and misconceptions about vaccination, low trust, household and gender dynamics, competing livelihood priorities, distance and transportation costs, inconvenient service hours, negative experiences with health services, and vaccine or health-worker availability [10,11]. However, these determinants are often studied separately or grouped into categories of barriers and facilitators. Such an approach helps identify what promotes or limits vaccination, but it explains less clearly how these different factors interact between the moment a family learns about a vaccination activity and the moment the child is actually vaccinated.

Several conceptual frameworks have contributed to a better understanding of vaccination behaviors. The Behavioural and Social Drivers of Vaccination (BeSD) model developed by WHO notably distinguishes thoughts and feelings, social processes, motivation, and practical issues that influence vaccination [12]. The COM-B model, for its part, considers behavior to result from the interaction between capability, opportunity, and motivation [13]. Other approaches, such as the Theory of Planned Behavior, emphasize the influence of attitudes, social norms, and perceived behavioral control on intentions and behaviors [14]. These models show that vaccination decision-making is multidimensional and cannot be reduced solely to knowledge of the benefits of vaccination.

In the specific context of catch-up vaccination, however, an important question remains: how do these different dimensions unfold or interact to lead to the effective participation of families? A parent may have received the information without having sufficiently understood it; may have understood the message without trusting its source; may accept the principle of vaccination while being influenced by their social environment; or may wish to have their child vaccinated without being available at the time when the service is offered. Non-participation may therefore result from different points of disruption within the same pathway. This distinction is particularly important for programmes, because these situations do not necessarily require the same interventions.

Previous research from peri-urban Abidjan has shown that childhood immunization is influenced by caregiver knowledge and sociodemographic characteristics [15]. However, the current catch-up context requires a more detailed understanding of how community, social and service-delivery factors interact to influence participation, particularly in densely populated urban and peri-urban settings.

A better understanding of participation therefore requires moving beyond an interpretation based on a succession of independent barriers to examine the relationships among these different dimensions. Such an approach can help identify the points at which the process leading to vaccination is strengthened or, conversely, interrupted, and thereby guide catch-up interventions more precisely.

This study aimed to analyze the dimensions associated with community participation in catch-up vaccination activities and their relationships in two peri-urban districts of Abidjan, Côte d’Ivoire. Drawing on the experiences and perceptions of parents, community actors, and professionals involved in vaccination, it proposes the community participation pathway for catch-up vaccination, an interpretive conceptual framework describing the pathway linking information about catch-up activities to the effective vaccination of the child.

## 2. Methods

### 2.1. Study Design and Setting

This analytical qualitative study was conducted between July and November 2025 in the peri-urban health districts of Abobo and Yopougon, in Abidjan, Côte d’Ivoire. It was part of a broader research project on the implementation of catch-up vaccination strategies for zero-dose and under-vaccinated children.

These two urban and peri-urban districts were selected because of their high population density and the substantial presence of under-vaccinated children.

The present analysis focused specifically on the dimensions of community participation in catch-up vaccination and on the relationships among these dimensions, from information about the activity to the effective vaccination of the child.

### 2.2. Study Population and Sampling

Purposive sampling was used to include participants directly involved in the planning, implementation, or use of catch-up vaccination services. Participants included health managers at regional and district levels, health facility professionals, community health workers (CHWs), community actors, and parents or caregivers of children under five years of age.

Professionals and community actors were selected according to their involvement in vaccination and community mobilization activities. Parents were recruited in the study areas to document their experiences and perceptions regarding catch-up activities.

In total, 106 participants were included: 26 took part in semi-structured individual interviews and 80 in eight focus group discussions, consisting of four groups of women and four groups of men, with ten participants in each group.

### 2.3. Data Collection

Data were collected between July and November 2025 through 26 semi-structured individual interviews (IIs) and eight focus group discussions (FGDs). The guides, developed in French by the INSP research team, explored perceptions and experiences related to catch-up vaccination, including information and community mobilization, perceptions and beliefs about vaccination, constraints faced by families, and the organizational conditions of the activities.

The individual interviews were conducted with professionals and actors involved in the planning or implementation of vaccination activities. Conducted by trained members of the research team using a semi-structured guide, they focused in particular on the organization of catch-up vaccination, community mobilization, implementation challenges, and factors likely to influence community participation. The interviews were recorded using a voice recorder and then fully transcribed. The study was reported in accordance with the Consolidated Criteria for Reporting Qualitative Research (COREQ) [16].

The FGDs brought together 80 parents or caregivers of children aged 0 to 59 months, divided into four groups of men and four groups of women, with ten participants in each group. They were conducted in French by trained members of the research team, assisted by note-takers, in community spaces that ensured confidentiality. The discussions, lasting approximately 60 min, were recorded with participants’ consent and supplemented by field notes [16].

The number and types of individual interviews and focus group discussions were predefined in the study protocol. Therefore, thematic saturation was not used as a formal stopping criterion for data collection. Nevertheless, data collection followed an iterative process, with debriefing sessions conducted after interviews and focus group discussions to identify emerging findings and issues requiring further exploration in subsequent data collection.

### 2.4. Analytical Approach

All individual interviews and focus group discussions were conducted in French, and no interpretation or translation was required during data collection. Audio recordings were transcribed in French, and coding and thematic analysis were conducted using the original French-language transcripts. Translation into English was performed only during manuscript preparation, including for the verbatim quotations reported in the manuscript.

Qualitative coding was conducted by three members of the research team using NVivo 14. A standardized thematic codebook containing operational definitions guided the systematic coding of the transcripts. Transcripts were distributed among the three coders, and consistency across coders was supported through the use of the same standardized codebook throughout the coding process. The thematic codebook, together with the semi-structured individual interview and focus group discussion guides, is provided in the Supplementary Materials.

Thematic analysis was used to identify recurring themes related to community participation in catch-up vaccination. Codes were grouped into thematic categories and compared across participant groups to examine convergences and divergences in their experiences and perceptions [17]. Particular attention was paid to relationships among themes, especially those related to information and community mobilization, perceptions and rumors about vaccination, social and practical constraints, the organization of activities, and the role of community actors. This cross-sectional analysis informed the dimensions used to construct the conceptual framework presented in the following section.

### 2.5. Construction of the Conceptual Framework

The construction of the conceptual framework was based on a cross-sectional analysis of the identified themes. Beyond grouping them into categories, the analysis examined relationships emerging from participants’ accounts between information about catch-up activities, understanding of messages, perceptions and trust in vaccination, social influences, parents’ availability, and the organizational conditions of the activities.

Convergences and divergences among the perspectives of parents, community actors, and professionals were examined in order to identify the dimensions operating between information about the catch-up activity and the effective vaccination of the child. These dimensions were then organized into an interpretive framework.

The resulting conceptual framework was developed as a qualitative conceptual pathway to map the sequential dependencies and potential disruption points identified in participants’ accounts. The arrows represent an interpretive organization of the pathway rather than quantitatively established causal relationships or a strictly deterministic sequence.

## 3. Results

### 3.1. Participant Characteristics

A total of 106 people participated in the study: 26 in semi-structured individual interviews and 80 in eight focus group discussions, divided into four groups of women and four groups of men, with ten participants in each group. The sociodemographic and professional characteristics of the participants are presented in Table 1.

Interview participants had a mean age of 47.2 years and a mean professional experience of 16.5 years. Men accounted for 65.4% of this group. Participants’ profiles covered several levels of the health system, from the regional level to community actors.

In the focus groups, the mean age was 40.6 years among women and 42.1 years among men. Women were more represented in petty trade, hairdressing, and sewing, while men were engaged mainly in trade, agriculture, and manual activities.

### 3.2. Dimensions of Participation in Catch-Up Vaccination

The cross-sectional analysis of the data identified several dimensions operating between information about catch-up activities and the vaccination of the child. Participants’ accounts highlighted exposure to information, understanding of messages, trust in actors and services, social acceptance, and parents’ availability and capacity, as well as their participation in activities. The effective vaccination of the child represented the endpoint of this pathway.

Linking these dimensions led to the development of the Community Participation Pathway for Catch-up Vaccination (CPP-CV), structured around seven components: exposure to information, understanding of the message, trust in actors and services, social acceptance, parent availability and capacity, participation in the activity, and effective vaccination of the child.

The analysis also identified five groups of cross-cutting factors likely to intervene at different stages of the pathway: community and health system actions; social, cultural, and religious norms; material and structural constraints; organization of activities; and characteristics of parents or caregivers. These factors included, in particular, the role of CHWs and community leaders, beliefs and social influences, distance and costs, service schedules and accessibility, vaccine availability, as well as parents’ level of education, previous experiences, and priorities.

**Figure 1:**
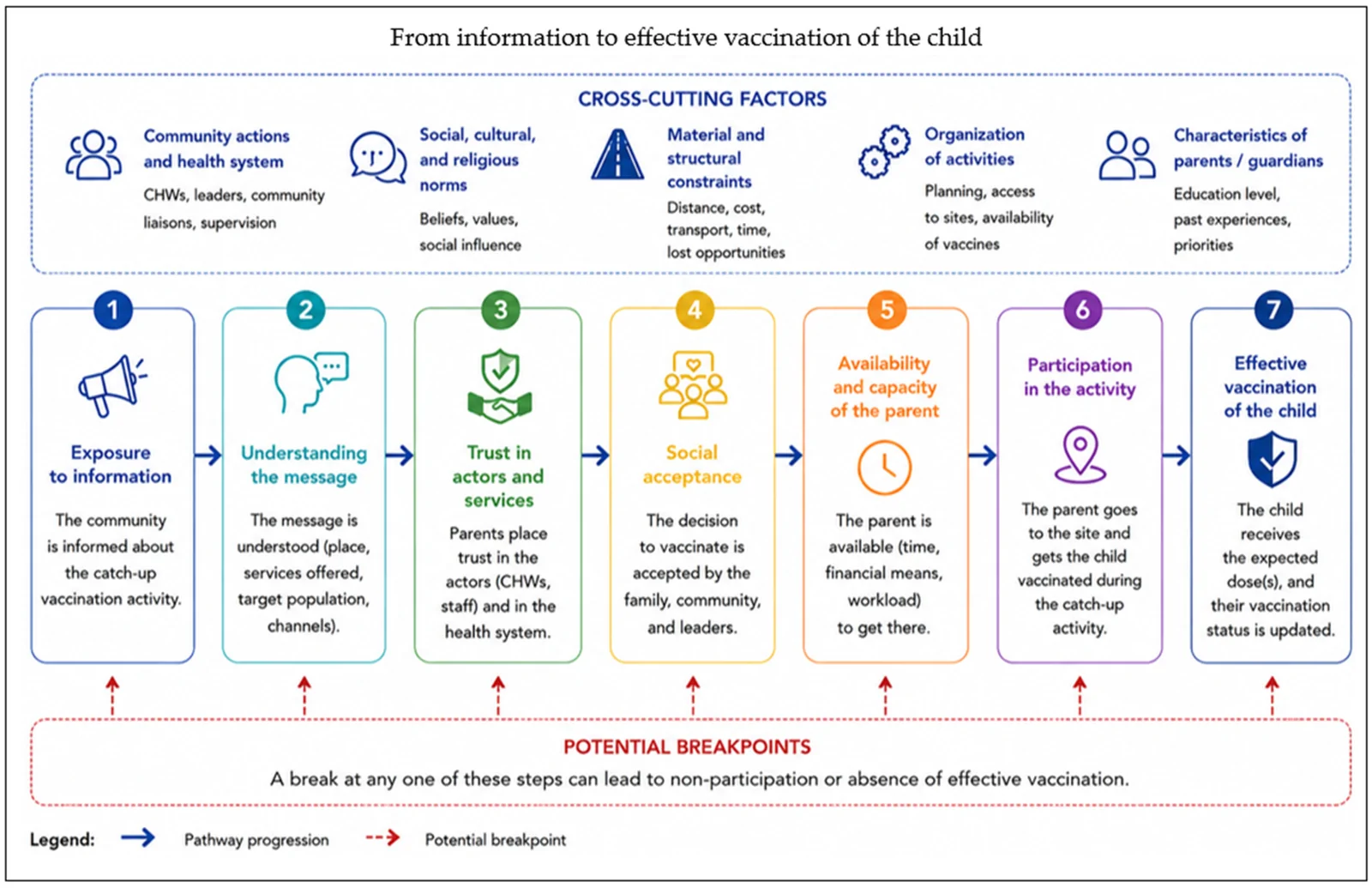
Conceptual framework of the Community Participation Pathway for Catch-up Vaccination (CPP-CV).

The seven components and cross-cutting factors of the CPP-CV are presented in Figure 1 and detailed in the following sections.

### 3.3. Exposure to Information

Lack of exposure to information was frequently reported. Several parents indicated that they learned about catch-up activities late, sometimes when vaccination teams arrived. One participant stated:

*“Regarding vaccination campaigns, we only find out when the health worker is already there. […] Even before the health worker arrives, we should be informed.”*

(FGD, participant 2, 40 years old)

The frequency and understanding of messages were also mentioned. Some participants felt that one-time information did not make it possible to reach all parents, particularly those who were absent from home:

*“The information is shared once. And it is not repeated. So, a father may not hear it.”*

(FGD, participant 3, 35 years old)

Different dissemination channels were used, including schools:

*“Often, even schools are involved; letters are distributed in schools, to school principals. They put small pieces of paper in the children’s bags to inform them that on such a day, health workers will come to vaccinate the child.”*

(II, participant 24, 53 years old)

Door-to-door visits, community radio, and the involvement of religious and community leaders were among the main mobilization methods cited. The experiences reported thus highlighted three dimensions of information: anticipation, repetition, and understanding.

### 3.4. Understanding of the Message

Beyond receiving information, several participants reported a need to better understand the nature and purpose of the vaccination being offered.

*“Parents should be better sensitized so that they can understand the reason for the vaccine above all.”*

(FGD, participant 2, 41 years old)

Understanding could also be limited by the way messages were delivered:

*“Sometimes, it is at night that the town crier makes noise; you do not even know what they are talking about […] you cannot even tell the difference in what he is saying. […] You yourself have not understood anything.”*

(FGD, participant 7, 44 years old)

### 3.5. Trust in Vaccination and Its Actors

Concerns about vaccination related in particular to perceived costs and rumors. Although EPI vaccines are free of charge, some parents associated vaccination with payments:

*“I experienced catch-up vaccination myself, but when free vaccination is provided […] they make you pay. As a result, it discourages parents.”*

(FGD, participant 8, 42 years old)

Rumors were also a recurring theme. Some participants reported statements portraying vaccines as dangerous or “poisoned,” while others directly linked these perceptions to lack of information.

*“The vaccine that is coming is poisoned.”*

(FGD, participant 2, 40 years old)

*“They said the vaccine is meant to put people to sleep; it is lack of information.”*

(FGD, participant 8, 42 years old)

These statements revealed three recurring concerns in community perceptions: the perceived cost of vaccination, rumors about vaccines, and lack of information.

### 3.6. Social Acceptance

Acceptance of vaccination varied across families and communities. Some refusals were reported, but they could sometimes be overcome through exchanges with vaccination actors:

*“The population accepts it and encourages us. […] There are a few households that refuse vaccination… But with arguments, we end up convincing them.”*

(II, informant 12, 44 years old)

Convinced religious and community leaders also took part in awareness-raising activities:

*“We conduct outreach visits… We go to churches, funerals, festive events…”*

(II, informant 12, 44 years old)

### 3.7. Parents’ Availability and Capacity

Professional and family constraints were frequently mentioned as limiting participation in catch-up activities. Vaccination schedules sometimes coincided with working hours, reducing the availability of some parents, particularly fathers.

*“As far as we fathers are concerned, there is no time.”*

(FGD, participant 3, 35 years old)

Being absent from home when vaccinators came by was also a difficulty:

*“Not everyone is at home every day.”*

(FGD, participant 6, 39 years old)

### 3.8. Participation in Catch-Up Activities

Some participants directly linked lack of information to their non-participation in vaccination activities:

*“Personally, honestly, I have never accompanied a child […] because I have honestly never received any information about it.”*

(FGD, participant 5, 51 years old)

Another participant stated:

*“You cannot go and accompany someone when you do not have the information.”*

(FGD, participant 8, 32 years old)

### 3.9. Effective Vaccination of the Child

The final dimension concerned the completion of vaccination after mobilization and use of services. Some actors reported an increase in service attendance following awareness-raising activities:

*“After our awareness-raising activities, parents and everyone else began attending the hospital.”*

(II, informant 7, 49 years old)

Vaccination could also be offered in nearby community spaces in order to reach children. One informant stated:

*“We vaccinate in front of mosques, churches, and in markets. And also, under the VACCIPHA programme, we vaccinated in pharmacies as well.”*

(II, informant 10, 38 years old)

## 4. Discussion

This study aimed to better understand the dimensions that structure community participation in catch-up vaccination activities and the relationships that may exist among them. The findings show that this participation does not depend on a single factor. Exposure to information, understanding of that information, trust in actors and services, social acceptance, parents’ availability and capacity, as well as the conditions enabling their participation, all contribute in complementary ways to the pathway leading to vaccination [6,7,8,9,10,11]. These observations led to the proposal of the Community Participation Pathway for Catch-up Vaccination (CPP-CV), an interpretive framework structured around seven components.

One of the main lessons concerns exposure to information. Participants frequently reported late and rarely repeated information, sometimes received at the very moment when vaccination teams arrived. This confirms the importance of communication but also shows that simply being exposed to a message does not guarantee participation. The timing, repetition, and understanding of the message appear equally important [9]. This finding is consistent with a recent systematic review of vaccination communication strategies across Africa, which highlighted the value of vaccine education, reminders, trust-building and community involvement and showed that community-based social mobilization can complement broader communication strategies [18]. Importantly, the present findings suggest that exposure to information and understanding of that information should be considered distinct steps: communication may reach a caregiver without necessarily generating sufficient understanding to support participation. The findings also highlight a lack of proactive and continuous information, as well as the sometimes-informal nature of information received by communities. The CPP-CV therefore distinguishes exposure to information from understanding of the message and trust in actors and services.

This interpretation is consistent with WHO’s Behavioral and Social Drivers of Vaccination (BeSD) framework [12], as well as the COM-B model [13], which respectively emphasize the role of perceptions, social processes, practical constraints, motivation, and opportunities. The CPP-CV does not seek to replace these models but rather proposes a sequential and operational interpretation applied to catch-up vaccination. In particular, it makes it possible to examine the point at which, between exposure to information and the effective vaccination of the child, participation is built or may break down.

The rumors and perceptions reported in the study reinforce this interpretation. Some communities associated vaccination with presumed risks or with payments perceived as inconsistent with the announced free-of-charge nature of vaccination. These elements can undermine the credibility of the message and the relationship with services. The literature also shows that vaccine confidence does not concern only the product itself, but also the professionals, institutions, and delivery mechanisms through which vaccination is offered [6,7,19]. In the CPP-CV, trust in actors and services therefore occupies an important position between understanding of the message and social acceptance, without being considered a demonstrated causal mechanism.

Social acceptance also appears to be influenced by the family and community environment. Certain cultural or religious beliefs could limit acceptance of vaccination, whereas the involvement of religious and community leaders was described as facilitating mobilization. This dual function is important: community actors should not be viewed only as potential sources of resistance, but also as intermediaries capable of legitimizing activities [8,20]. This observation is consistent with socio-ecological approaches and the Theory of Planned Behavior [14,20], which emphasize the importance of social norms and the interpersonal environment in shaping behaviors.

The results also show that a favorable attitude toward vaccination does not guarantee effective participation. Professional, family, and time-related constraints reduced the availability of some parents, particularly in households dependent on informal activities. This distinction between social acceptance and parents’ availability and capacity is essential. It avoids interpreting every absence as vaccine hesitancy or refusal. In some cases, the parent may wish to vaccinate the child but may not have the time, resources, or transportation options needed to participate in the activity [12,13].

This distinction justifies the place of parents’ availability and capacity as a specific component of the CPP-CV. It also implies that programmatic responses may vary according to the point of disruption identified. Information-related difficulty calls for earlier and repeated communication; acceptance-related difficulty may require the involvement of credible community actors; a practical constraint may be better addressed through adapted schedules, outreach strategies, or improved service accessibility [13,21].

Beyond the seven components, the pathway is influenced by several cross-cutting factors that may intervene at different stages [22]. The CPP-CV groups them into five categories: community and health system actions; social, cultural, and religious norms; material and structural constraints; organization of activities; and characteristics of parents or caregivers. These factors make it possible to consider, in particular, the role of CHWs and leaders, beliefs and social influences, distance, cost, and transport constraints, service accessibility and availability, as well as parents’ previous experiences and priorities [23].

The organizational conditions of activities constitute, in this respect, an important dimension of the pathway environment [24]. Participants reported difficulties related to human resources, logistics, access to certain areas, and the working conditions of CHWs [25]. Lack of resources may limit mobilization and access to areas where zero-dose or under-vaccinated children live. Progression toward vaccination therefore does not depend exclusively on parents; it also depends on the health system’s capacity to provide a genuinely accessible vaccination opportunity.

CHWs particularly illustrate this interface between the community and the health system. They contribute to information dissemination, mobilization, and bringing families closer to services, but their action may be limited by the size of the areas covered, the resources available, and motivational conditions. The findings therefore show that their ability to play this role also depends on the operational conditions under which they work.

Finally, the CPP-CV distinguishes participation in the activity from effective vaccination of the child [26]. This distinction is important because the parent’s movement to or presence at the activity represents one stage of the pathway, whereas its endpoint corresponds to the actual administration of the vaccine or vaccines needed by the child. Site accessibility, vaccine availability, and the organization of activities may therefore continue to influence the pathway up to this final stage. This distinction prevents community participation from being automatically equated with effective vaccination.

The main value of the CPP-CV therefore lies in the articulation of these components and the cross-cutting factors that influence them. Exposure to information opens the pathway; understanding of the message and trust in actors and services influence the way information is received; the social environment contributes to its acceptance; parents’ availability and capacity determine the practical possibility of participating; and participation can lead to effective vaccination when service delivery conditions allow it. The model should nevertheless be understood as an interpretive framework rather than as a rigid causal sequence.

The main contribution of this study is to show that non-participation is not necessarily a barrier in itself but may correspond to a disruption at different points along the pathway between information and effective vaccination. Identifying the potential point of disruption makes it possible to move from a uniform response to an implementation strategy that is better adapted to the mechanism involved. This articulation between pathway stages, disruption points, and cross-cutting factors constitutes the main conceptual added value of the CPP-CV.

Although the CPP-CV was developed specifically in the context of catch-up vaccination, several of its components may also be relevant to routine immunization. Information exposure, message understanding, trust, social acceptance, and parents’ availability and capacity are not necessarily specific to catch-up activities and may influence participation in routine vaccination services. However, catch-up vaccination has a distinct operational context, as it requires identifying and reaching children who have already missed scheduled vaccine doses and facilitating their re-engagement with vaccination services. For this reason, the framework is referred to as the Community Participation Pathway for Catch-up Vaccination (CPP-CV), reflecting the empirical context in which it was developed. Further research is needed to determine whether the framework can be transferred or adapted to routine immunization settings.

### Strengths and Limitations of the Study

This study has several strengths, including the diversity of participants and the combined use of individual interviews and focus group discussions, which made it possible to compare the perspectives of professionals, community actors, and parents. It nevertheless has limitations. The data come from two peri-urban districts of Abidjan, which may limit the transferability of the findings to other contexts. In addition, the relationships proposed in the CPP-CV are derived from a qualitative analysis and do not make it possible either to measure the relative weight of each component or to establish causality.

The CPP-CV should therefore be considered as a conceptual proposition to be tested and refined. Quantitative or mixed-methods studies could examine the relationships among its different components and assess its transferability to other contexts. From a programmatic perspective, the framework suggests that improving catch-up vaccination does not only mean providing more information, but also ensuring that messages are understood and credible, that the social environment is favorable, that parents have the practical capacities needed to participate, and that services make it possible to transform this participation into effective vaccination.

## 5. Conclusions

This study shows that community participation in catch-up vaccination does not depend on an isolated determinant, but on the articulation of several dimensions. Exposure to information constitutes the entry point of the pathway, but it is not sufficient to ensure vaccination of the child. Understanding of the message, trust in actors and services, social acceptance, parents’ availability and capacity, and participation in the activity all intervene before effective vaccination. These stages are also influenced by community, social, material, organizational, and individual factors. The Community Participation Pathway for Catch-up Vaccination (CPP-CV) makes it possible to organize these dimensions into a pathway from information to effective vaccination and to identify potential points of disruption.

From a programmatic perspective, immunization programmes should move beyond information dissemination alone and tailor interventions to the mechanisms underlying non-participation. This requires combining effective communication and trusted community engagement with flexible and accessible service delivery approaches that address parents’ practical constraints. Such context-specific and community-responsive strategies may help translate community awareness into effective vaccination and improve the reach of children who have been missed by routine immunization services.

The CPP-CV nevertheless remains an interpretive framework derived from qualitative data and not a demonstrated causal model. Its validation in other contexts and through quantitative or mixed-methods approaches will help clarify its robustness and usefulness for vaccination programmes.

## Figures and Tables

**Table 1:** Sociodemographic and professional characteristics of participants.

Characteristics	Individual Interviews (*n* = 26)	FGD Women (*n* = 40)	FGD Men (*n* = 40)
Number of participants, *n*	26	40	40
**Mean age**, years (SD)	47.2 (10.7)	40.6 (13.2)	42.1 (12.6)
**Sex**, *n* (%)			
Male	17 (65.4)	0 (0.0)	40 (100.0)
Female	9 (34.6)	40 (100.0)	0 (0.0)
Education level, *n* (%)			
No formal education	0 (0.0)	9 (22.5)	1 (2.5)
Primary education	2 (7.7)	14 (35.0)	12 (30.0)
Secondary education	10 (38.5)	12 (30.0)	16 (40.0)
Higher education	14 (53.8)	5 (12.5)	11 (27.5)
Mean professional experience, years (SD)	16.5 (11.1)	N/A	N/A

N/A, not applicable; professional experience was not applicable to focus group participants.

## Data Availability

The coding framework and thematic codebook developed using NVivo 14^®^ may be obtained from the corresponding author upon reasonable request, subject to institutional data-sharing agreements. Anonymized qualitative data cannot be made publicly available because of confidentiality commitments made to participants.

## Supplementary Materials

The following supporting information can be downloaded at: https://www.mdpi.com/article/10.3390/vaccines14090786/s1, S1: CODEBOOK PROJET PEV_DEMANDE_2025; S2: Focus Group Guide—MEN in rural areas; S3: Focus Group Guide—WOMEN in rural areas; S4: Interview Guide—Community Leaders in Rural Areas; S5: Interview Guide—DCPEV and EPI Partners; S6: Interview Guide—District Manager; S7: Interview Guide for the technical actors of the health district; S8: Interview Guide for Nurse, Midwife & CHW.

## Abbreviations

DCPEV	Directorate for the Coordination of the Expanded Programme of Immunization
EPI	Expanded Programme of Immunization
FGD	Focus Group Discussion
IA2030	Immunization Agenda 2030
II	individual interview
INSP	National Institute of Public Health
MSHPCMU	Ministry of Health, Public Hygiene and Universal Health Coverage
UHC	Universal Health Coverage
SD	Standard Deviation
