# Supplementary material for: From Information to Vaccination: A Conceptual Framework for Understanding Community Participation in Catch-Up Vaccination for Children in Abidjan, Côte d’Ivoire
Source: Vaccines (Basel). 2026 Sep 8;14(9):786. doi: 10.3390/vaccines14090786 (PMC13612099; doi:10.3390/vaccines14090786)
Supplement: Supplementary file 1 [file vaccines-14-00786-s001.zip › S1. CODEBOOK PROJET PEV_DEMANDE_2025.pdf]

REPUBLIQUE DE CÔTE D'IVOIRE
Union – Discipline – Travail

MINISTERE DE LA SANTE DE L'HYGIENE PUBLIQUE ET DE LA COUVERTURE MALADIE UNIVERSELLE

INSTITUT NATIONAL DE SANTE PUBLIQUE

RECHERCHE SUR LA MISE EN ŒUVRE DE LA VACCINATION EN CÔTE D'IVOIRE

Volet 2 : Demande de vaccination

I. Codification des fichiers audios et Word

Chaque fichier audio et document Word sera nommé en liant les informations suivantes

Type d'entretien, la structure sanitaire, répondant, le lieu où se situe l'établissement et la région,

Les différentes parties du code seront séparées par le tiret du huit. _

Ainsi nous aurons : Entretien individuel_structure sanitaire_ lieu de l'établissement _Région Répondant_

EXEMPLE : EI_CSR_ KOUASSI N'DAWA _BOND_LCom

Pour les cibles, les abréviations suivantes seront utilisées dans le code :

- Coordonnateur du PEV = CPEV
- Leadeur communautaire = LCom
- Agent de Santé communautaire = ASC
- Responsable de la vaccination = RPEV
- Responsable de région = DR, DD, CSA

Pour les régions, les abréviations suivantes seront utilisées dans le code :

Abidjan = ABID
Abobo Est = ABO.E
Abobo Ouest = ABO.O
Yopougon Est = YOP.E
Yopougon Ouest = YOP.O
Agboville = AGBO
Yamoussoukro = YAMO
Tiébissou = TIEB
Sikensi = SIKE
San Pedro = SANP
Tabou = TABO
Blolequin = BLOL
Guiglo = GUIG
Sinematiali = SINE
Korhogo = KORH
Bondoukou = BOND
Tanda = TAND

II. Règles d'encodage

L'encodage consistera à lire les entretiens et appliquer le code correspondant à un bout de phrase.

L'encodage suivra les principes suivants :

- Toutes les portions de textes doivent être encodées : appliquer le nœud 0. Aucun nœud lorsque le passage ne renvoie à aucun nœud. ,
- Dans le processus d'encodage, l'agent s'intéresse plus aux réponses des enquêtés qu'aux questions posées. En effets, les réponses ne sont pas toujours en lien direct avec

les questions posées, mais elles ne sont pas pour autant sans intérêt, car pouvant comporter des thématiques retenues pour cette étude.

- Sélectionner une partie assez large de texte qui permet de comprendre l'idée qui est développée.
- Lorsque les réponses sont trop courtes pour que l'on comprenne de quoi il s'agit, vous devez sélectionner aussi la question qui a été posée.
- Un nœud peut être appliqué autant de fois qu'il apparait dans le texte.
- Une même portion de texte peut contenir plus d'un thème ; dans ce cas vous appliquerez tous les nœuds concernés.

III. Caractéristiques des sources

N.B. Elles seront affectées par les responsables de l'analyse de chaque régions. .

Identification

Pour chaque entretien, les caractéristiques doivent être précisées dans un fichier Excel. Les modalités de chaque caractéristique sont décrites dans le tableau ci-dessous.

Les règles suivantes doivent être respectées pour mettre une importation directe dans le logiciel Word

Les codes des entretiens dans le fichier Excel et des entretiens importés doivent être identiques

IV. Description des thèmes et sous thèmes

THEMES	Sous thème1	Sous thème2	Codes	Description
Aucun nœud				
CONNAISSANCES	STRATÉGIES DE SENSIBILISATION ET RECHERCHE DES ENFANTS ZÉRO DOSE ET SOUS VACCINÉS	QD1-1 : Normes, directives et autres documents, nationaux existants	CSNN_normes, directives, ZD, SV	Normes, directives et autres documents, nationaux existants pour l'amélioration de la stratégie de sensibilisation et de recherche des enfants Zéro Dose (ZD) et sous vaccinés (SV). Au niveau régional ou du district
		QD1-2 : Accessibilité, Normes, directives et autres documents, nationaux existants	CSAN_accessibilité, Normes, directives, structure de santé	Accessibilité, des documents, (normes et directives)
		QD1-3 : Utilisation des données pour amélioration des stratégies,	CSUA_utilisation données, amélioration stratégie, ZD, SV	Utilisation de données collectées au niveau des structures de santé et dans la communauté en vue de prendre des décisions pour l'amélioration des stratégies de sensibilisation et recherche des enfants Zéro Dose (ZD) et sous vaccinés (SV)
		QD1-4 : Existence de rapports entre la direction de santé, les ONG, la communauté et autres structures intervenant dans le domaine de la vaccination	CSER_rapports, domaine Vaccination	Rapports existants entre la direction de santé, les ONG, la communauté et autres structures intervenant dans le domaine de la vaccination de votre territoire
		QD1-5: Existence de politique de délégation des taches de la demande vaccinale	CSED_politique délégation, demande vaccinale	Existence de politique de délégation des taches au niveau de la demande vaccinale

		QD1-5: Le paquet d'activités des ASC	CSCAA, composition, activité, ASC	Quelle est la composition du paquet d'activités des ASC ?
	**ACTEURS DE LA VACCINATION**	QAT1-1 : Stratégie de sensibilisation et recherche des enfants ZD et SV	CAVSR_ Activités, stratégie de sensibilisation, recherche ZD et SV	Activités menées qui sont en rapport avec la stratégie de sensibilisation et recherche des enfants Zéro Dose (ZD) et sous vaccinés (SV).
		QAT1-2 : Enumération des activités menées avec les agents de santé	CAVEA_Activités, aire sanitaire	Enumération des activités menées avec les agents de santé (ASCs, IDE, SFDE, médecin) dans l'aire sanitaire.
		QISA1-2 : Activités des ASC	CAAMA_activités menées, ASC	Activités menées avec les agents de santé communautaire de l'aire sanitaire.
		QISA1-1 : Vaccins disponibles pour la Vaccination des enfants	CAVPE_vaccins proposés, enfants	Vaccins proposés pour la vaccination des enfants dans l'aire de santé.
		QISA1-3 : Fréquentation du centre de santé par les populations.	CAFSP_utilisation, centre de santé, population	Utilisation fréquente du centre de santé par les populations.
		QISA1-4 : Raisons de la fréquentation du centre de santé par la population	CARFS_raisons, populations, centre de santé	Raisons évoquées par les populations pour la fréquentation du centre de santé.
		QISA1-5-1 : Raisons de la non fréquentation du centre de santé par la population	CARNF_raisons, non fréquentation, centre de santé.	Raisons de la non fréquentation du centre de santé.
		QAT1-3 : Enumération des raisons pour l'entreprise de stratégies de sensibilisation, et recherche de ZD et SV	CAERAEnumération raisons, stratégie de sensibilisation, recherche ZD et SV	Enumération des raisons qui amènent les agents de santé (ASCs, IDE, SFDE, médecin) à entreprendre des stratégies de sensibilisation et recherche des enfants Zéro Dose (ZD) et sous vaccinés (SV).

		QISA1-5-2 : Raisons de la non fréquentation du centre de santé par la population	CARNF_raisons, non fréquentation, centre de santé.	Raisons de la non fréquentation du centre de santé.
**PLANIFICATION ET COORDINATION**		QDP1-1 : Existence d'un plan stratégique national ou des Directives spécifiques	PCEPD_Existence, plan stratégique national, directives spécifiques, rattrapage vaccinal	Existence d'un plan stratégique national ou des directives spécifiques encadrant le rattrapage vaccinal des enfants ZD et SV
		QDP1-2 : Contribution de l'institution à la conception ou à la mise en œuvre de ce plan stratégique	PCCCP_Contribution, conception, plan stratégique	Contribution de l'institution à la conception ou à la mise en œuvre de ce plan stratégique
		QDP1-3 : Contextualisation de La planification au niveau local	PCCPL_Contextualisation, planification, local	Contextualisation de La planification au niveau local (districts, communautés rurales)
		QDP1-5-1 : Association des populations à la planification	PCAPP_Association, populations, planification	Association des populations ou leurs représentants à la planification
		QDP1-5-2 : Raisons de la non association des populations à la planification	PCRNP_Raisons, non association, populations, planification	Raisons de la non association des populations ou leurs représentants à cette planification
		QD2-1 : Existence de plan opérationnel	PCEPO_ plan opérationnel, district, rattrapage vaccinal.	Existence de plan opérationnel au niveau du district pour la mise en œuvre de la stratégie de rattrapage vaccinal.
		Méthode d'élaboration du plan opérationnel	PCMPO_ élaboration, plan opérationnel, rattrapage, vaccinal, acteurs	Méthode d'élaboration du plan opérationnel pour la mise en œuvre de la stratégie de rattrapage vaccinal et les acteurs inclus dans ce processus

		Existence de plan local de mise en œuvre de la stratégie de sensibilisation et de rattrapage vaccinal.	PCEPL_plan local, stratégie de sensibilisation, rattrapage vaccinal.	Existence d'un plan local de mise en œuvre de la stratégie de sensibilisation et de rattrapage vaccinal.
		Rôle de l'acteur dans l'élaboration du plan de mise en œuvre local.	PCRPM_ Rôle, élaboration, plan MEO	Rôle dans l'élaboration du plan de mise en œuvre local.
		QDP1-4 : Méthodes utilisées pour organiser la coordination entre les différents niveaux	PCMCN_Méthodes, coordination, niveaux	Méthodes utilisées pour organiser la coordination entre les différents niveaux (national, régional, local)
		QD2-3 : Méthodes mise en place pour assurer la coordination entre les structures de santé, les partenaires et les communautés.	PCMS_méthodes, oordination,	Méthodes mise en place pour assurer la coordination entre les structures de santé, les partenaires et les communautés.
		Description du suivi de la mise en œuvre.	PCDSM_ Description, suivi	Description du suivi de la mise en œuvre.
		Harmonisation de la stratégie	PCHSI_Harmonisation, stratégie, autres intervenants	Harmonisation de la stratégie avec les autres intervenants de santé.
		Présentation des synergies existant entre les composantes PEV, suivi-évaluation et communication	PCSCS_synergies, composantes PEV, suivi-évaluation, communication	Présentation des synergies existant entre les composantes PEV, suivi-évaluation et communication.
		Niveaux d'implications dans la planification et la coordination des activités.	PCNIP_implications, planification, coordination	Niveaux d'implications dans la planification et la coordination des activités.

		Participation de la population aux activités	PCAPA_association, activité	Association des populations de la localité à l'activité.
		QDP1-6 : principales contraintes rencontrées dans la coordination multisectorielle	PCECM_Enumération, contra intes, multisectorielle	Enumération des principales contraintes rencontrées dans la coordination multisectorielle
		QD-4 : Difficultés rencontrées dans la coordination	PCDCS_difficultés, coordination stratégie	Difficultés rencontrées dans la coordination de la stratégie
**SENSIBILISATION**	**COMMUNAUTAIRE**	Définition des approches de communication	SCDAS_ Définition, communication, stratégie	Définition des approches de communication mises en œuvre pour accompagner la stratégie.
		Stratégies de communication mises en place au niveau national et local	SCSCN_Stratégies, communication, national, local, rattrapage	Stratégies de communication mises en place au niveau national et local pour accompagnement de la stratégie de rattrapage vaccinal des enfants ZD et SV
		Stratégie de communication adaptée au contexte local.	SCSCL_ Stratégie , communication, contexte local	Mis en place de stratégie de communication adaptée au contexte local.
		Méthodes utilisées pour l'implication des relais communautaires	SCMIR_Méthodes, implication, relais Communautaires	Méthodes utilisées pour l'implication des relais communautaires dans la sensibilisation.
		Méthodes d'organisation de la sensibilisation au niveau du district.	SCMOD_méthodes, sensibilisation, district.	Méthodes utilisées pour organiser des actions de sensibilisation au niveau du district.
		Initiateurs ou coordonnateurs des actions de sensibilisation	SCICA_ Initiateurs, coordonnateurs, sensibilisation	Initiateurs ou coordonnateurs des actions de sensibilisation.
		Partenaires ou acteurs communautaires mobilisés	SCPAM_partenaires, acteurs, Sensibilisation	Partenaires ou acteurs communautaires mobilisés pour la sensibilisation.

		Sollicitation à participer aux campagnes d'information ou de sensibilisation	SCSCI_ Sollicitation, campagnes d'information, de sensibilisation	Sollicitation à participer aux campagnes d'information ou de sensibilisation sur la vaccination;
		Types d'activités de sensibilisation organisés	SCTAS_Types, activités, Sensibilisation	Types d'activités de sensibilisation qui ont été organisés (radios, campagnes, mobilisation sociale, etc.)
		Actions de sensibilisation mises en œuvre.	SCAMO_actions, sensibilisation	Actions de sensibilisation mises en œuvre.
		Chargés de la sensibilisation communautaire.	SCPCS_personnes, chargés, sensibilisation communautaires	Les personnes ou association chargés de la sensibilisation communautaire.
		Processus d'implication des relais communautaires et des leaders locaux dans ces actions de sensibilisation	SCPIS_Processus, implication, relais communautaires, leaders locaux, sensibilisation	Processus d'implication des relais communautaires et des leaders locaux dans ces actions de sensibilisation.
		Acteurs impliqués	SCAIS_Acteurs, Sensibilisation	Catégories d'acteurs impliqués dans la diffusion des messages (agents, matrones, voisines, etc.).
		Rôle dans ces campagnes.	SCRC_Rôle, campagnes.	Rôle dans ces campagnes.
		Participation des femmes	SCRPF_Participation, Femmes	Rôle et participation possible des femmes dans les actions de sensibilisation.
		Enumération des actions de sensibilisation	SCEAL_Enumération, sensibilisation	Enumération des actions de sensibilisation organisées dans la localité ces derniers mois.
		Types d'outils et supports élaborés	SCTOE_Types, outils, supports, élaborés	Types d'outils et supports élaborés par la DCPEV ou ses partenaires.
		Elaboration d'outils et supports adaptés aux contextes locaux	SCAOC_Adaptation, outils, supports, contextes locaux	Adaptation des outils et supports élaborés par la DCPEV ou ses partenaires aux

				contextes locaux (langues, coutumes, canal privilégié) ?
		Production ou adaptation de supports spécifiques	SCPAS_supports spécifiques	Production ou adaptation de supports spécifiques.
		Existence de canaux plus efficaces	SCECE_canaux plus efficaces	Existence de canaux plus efficaces.
		Canaux efficaces pour toucher la population	SCCEP_Canaux, population	Canaux efficaces pour toucher la population locale.
		Canaux de sensibilisation de la population	SCCSP_canaux, populations	Canaux privilégiés pour toucher les populations
		Formation ou une orientation reçue pour participer aux actions sensibilisation	SCFRA_Formation, sensibilisation	Formation ou une orientation reçue pour participer aux actions
		Fréquence des actions de sensibilisation communautaire réalisée par la DCPEV ou ses partenaires	SCFAS_Fréquence, sensibilisation	Fréquence des actions de sensibilisation communautaire réalisée par la DCPEV ou ses partenaires.
		Fréquence de la réalisation de la sensibilisation	SCFRS_fréquence, sensibilisation communautaire	Fréquence de la réalisation de la sensibilisation communautaire.
		Fréquence des actions de sensibilisation.	SCFAS_Fréquence, sensibilisation.	Fréquence des actions de sensibilisation.
		Fréquence des actions	SCFAS_Frèquence, Actions	Fréquence à laquelle les campagnes de sensibilisation ont lieu.
		Formes de sensibilisation	SCFSF_Formes, Sensibilisation	Formes de sensibilisation les plus marquantes pour les femmes (rencontres, radios, animations, etc.).
		Information reçue	SCIRS_Info, Sensibilisation	Modes d’information sur les activités de vaccination et de sensibilisation.

		Compréhension du message	SCCM_Compréhension, Message	Niveau de compréhension du message transmis lors des campagnes.
		Activités de sensibilisation	SCAPC_activités perçus	Activités de sensibilisation perçues par les communautés
		Difficultés rencontrées dans la mobilisation	SCDMP_Difficultés, mobilisation	Difficultés rencontrées dans la mobilisation des populations.
		Evaluation des actions menée	SCEAM_évalation, actions	Existence d'évaluation de la portée des actions menées
		Dénombrement des évaluations ou des retours d'impact sur ces campagnes de sensibilisation.	SCDES_Dénombrement, évaluations, sensibilisation.	Dénombrement des évaluations ou des retours d'impact sur ces campagnes de sensibilisation.
	recherche des enfants ZD et SV	Types d'activités de sensibilisation	SRTAM_Types, Activités	Formes d'activités menées (réunions, annonces radio, visites à domicile, etc.).
		Fréquence des actions	SRFAM_Frèquence, Activités	Fréquence à laquelle se tiennent les actions de sensibilisation.
		Acteurs impliqués	SRAM_Acteurs, Sensibilisation	Catégories d'acteurs mobilisés (agents de santé, leaders communautaires, ONG).
		Efficacité perçue	SREPS_ Appréciation , Efficacité, Sensibilisation	Appréciation de l'efficacité des activités selon les participants.
		Rôle communautaire	SRRC_Rôle, Communauté	Rôle des pères/gardiens ou autres membres dans les activités de sensibilisation.
**RECHERCHE ACTIVE**		Recherche active, composante intégrée à la stratégie	RACS_composante, stratégie.	Recherche active, comme une composante intégrée à la stratégie.
		Prevention de phase de recherche active au niveau du district.	RAPRD_prevention, district.	Prevention d'une phase de recherche active au niveau du district.

		Intégration de la stratégie de recherche active dans le plan de rattrapage.	RAIRA_Intégration, recherche active.	Intégration de la stratégie de recherche active (porte-à-porte, visites à domicile) dans le plan de rattrapage.
		Détermination de directives pour l'intégration de la recherche active	RADIR_Directives, intégration, recherche active	Détermination de directives émises pour l'intégration de la stratégie de recherche active (porte-à-porte, visites à domicile) dans le plan de rattrapage.
		Stratégies pour cibler les ménages avec enfants ZD ou SV	RASCM_ Existence stratégies, enfants ZD ou SV	Existence de stratégies pour cibler les ménages avec enfants ZD ou SV.
		Détermination des critères pour l'identification des ZD et SV.	RACIE_ critères, Identification, ZD et SV	Détermination des critères recommandés pour l'identification des enfants ZD et SV.
		Indentification enfants non ou sous vaccinés par des visites à domicile	RAIEV1_indentification enfants, visites à domicile	Identification des enfants non ou sous vaccinés par des visites à domicile
		Outils ou méthodes préconisés pour identification de la cible.	RAOMI_outils, méthodes, identification, enfants cibles	Outils ou méthodes préconisés pour identification des enfants cibles.
		Diffusion d'outils ou instruments spécifiques pour appuyer l'activité	RADOA_Diffusion, outils spécifiques, activité	Diffusion d'outils ou instruments spécifiques pour appuyer l'activité (listes, carnets, fiches)
		Possession d'outils ou supports pour l'orientation de la recherche active.	RAOSO_outils, supports, recherche active.	Possession d'outils ou supports pour l'orientation de la recherche active.
		Outils produits ou adaptés	RAOPA_outils	Outils produits ou adaptés pour cette étape.
		Implication d'acteurs non étatiques dans la recherche active des enfants ZD et SV.	RAIAR_Implication, acteurs , recherche active, ZD et SV.	Implication d'acteurs non étatiques (ONG, volontaires, sociétés civiles) dans la recherche active des enfants ZD et SV.
		Formation du personnel	RAFP_formation, Personnel, recherche active	Formation du personnel sur la recherche active.

		Perceptions des activités de la recherche active par les communautés	RAPAR_ Perceptions, communautés	Perceptions des activités de la recherche active par les communautés.
		Principales difficultés rencontrées	RADR_ difficultés rencontrées	Principales difficultés rencontrées.
		Evaluation de l'efficacité de la recherche active.	RAEER_ évaluation, efficacité, recherche active	Comment évaluation de l'efficacité de la recherche active.
		Evaluation de la qualité des données collectées	RAEQD_ données	Evaluation de la qualité des données collectées.
		Défis ou limites dans l'opérationnalisation	RADLO_ défis, limites	Constats de défis ou limites dans l'opérationnalisation.
**RATTRAPAGE VACCINAL**		Orientations , techniques données pour l'organisation des séances	RVOOS_Orientations, organisation, séances.	Orientations techniques données pour l'organisation des séances.
		Participation à l'élaboration de la cartographie ou au micro plan.	RVEMP_ élaboration, micro plan.	Participation à l'élaboration de la cartographie ou au micro plan.
		Documentation de l'approche nationale pour l'organisation des séances mobiles ou avancées	RVDAS_Documentation, approche nationale, organisation, séances	Documentation de l'approche nationale pour l'organisation des séances mobiles ou avancées (zonage, fréquence, ciblage).
		Suivi des performances des équipes mobiles	RVSPE_performances, équipes mobiles	Suivi des performances des équipes mobiles.
		Argumentation de la notification d'écarts entre le planning et la réalité sur le terrain.	RVEPT_écarts, planning, Réalité	Notification d'écarts entre le planning et la réalité sur le terrain. Argumentation.
		Mobilisations des moyens humains, logistiques et financiers.	RVMHL_mobilisations, moyens humains, logistiques, financiers	Mobilisations des moyens humains, logistiques et financiers.
		Détermination des moyens mobilisés pour les séances de rattrapage vaccinal	RVDMS_Détermination, moyens, séances	Détermination des moyens (financiers, humains, logistiques) mobilisés pour appuyer les séances de rattrapage vaccinal.

		Fréquences des séances avancées ou mobiles	RVFS_fréquences, séances Avancées ou mobiles	Fréquences des séances avancées ou mobiles fait pour le rattrapage vaccinal.
		Fréquence de sorties pour organiser le rattrapage vaccinal.	RVFSR_Fréquence sorties, rattrapage vaccinal.	Fréquence de sorties pour organiser le rattrapage vaccinal.
		Organisation de séances	RVOS_Organisation, séances mobiles ou avancées	Organisation de séances mobiles ou avancées dans votre aire de santé.
		Critères de la mise en œuvre	RVCMO_critères, mise en Œuvre	Critères guidant leur mise en œuvre.
		Responsable de la mise en œuvre	RVRMO_Responsable, rattrapage	Responsable de la mise en œuvre.
		Processus utilisés pour le suivi et le reporting des activités du rattrapage vaccinal.	RVPSR_processus, suivi, Reporting	Processus utilisés pour le suivi et le reporting des activités du rattrapage vaccinal.
		Participations des parents aux séances	RVPPR_Participations, parents, rattrapage vaccinal.	Participations des parents aux séances.
		Connaissances relatives aux séances de vaccination mobile ou avancée	RVCSV_ Connaissances, séances, vaccination mobile, avancée	Connaissances relatives aux séances de vaccination mobile ou avancée
		Méthodes d'organisation des séances de vaccination mobile ou avancée.	RVMOV_ Méthodes, séances, vaccination mobile, avancée.	Méthodes d'organisation des séances de vaccination mobile ou avancée.
		Participation à l'élaboration du plan stratégique	RVPEP_Participation, élaboration plan stratégique	Participation/association à l'élaboration du plan stratégique local.
		Processus de priorisation des localités	RVPPL_Processus, priorisation, localités	Processus de priorisation des localités
		Choix des lieux et des dates	RVCLD_ Choix, lieux, dates	Choix des lieux et des dates
		Informations reçues avant le déroulement des séances	RVIS_ Informations,	Informations reçues avant le déroulement des séances

		Participation à la planification des séances	RVPPS_Participation, planification, séances	Participation à la planification des séances
		Rôle dans l'accueil ou la mobilisation de la population pour ces séances	RVRAM_Rôle, accueil, mobilisation	Rôle dans l'accueil ou la mobilisation de la population pour ces séances
		Suivi des séances par les familles	RVSSF_Suivi, séances, familles	Suivi des séances par les familles
		Enumération des difficultés rencontrées	RVRDR_Enumération, difficultés	Enumération des difficultés rencontrées (adhésion communautaire, accessibilité géographique, logistique) lors du rattrapage vaccinal.
		Raisons du non suivi des séances	RVRNS_ Raisons, non-suivi	Raisons du non suivi des séances
		Propositions pour l'amélioration des campagnes	RVPAC_Propositions, amélioration, campagnes	Propositions pour l'amélioration des campagnes dans la communauté
		Existence d'enfants qui échappent à la vaccination	RVEEV_Existence, enfants, Vaccinatiion	Existence, enfants qui échappent à la vaccination
		Raisons de la non vaccination des enfants	RVRNV_Raisons, non vaccination, enfants	Raisons de la non vaccination des enfants
		Détermination d'indicateurs de suivi et d'évaluation	RVDIR_Détermination, indicateurs,	Détermination d'indicateurs de suivi et d'évaluation du rattrapage vaccinal si existence de stratégie de suivi-évaluation de ces séances.
	recherche des enfants ZD et SV	Organisation des séances	RVROS_Organisation, Séances	Lieux, modalités et fréquence des séances de rattrapage vaccinal.
		Information et communication	RVRIC_Info, Communication	Sources d'information des familles concernant les séances.

		Participation aux séances	RVRPS_Participation, Séances	Participation effective des femmes aux séances de vaccination (mobiles ou avancées).
		Information sur les séances	RVRIS_Info, Séances	Canaux par lesquels les femmes sont informées des séances de vaccination.
		Accessibilité des lieux	RVRAL_Accessibilité, Lieux	Facilité ou difficulté d'accès aux lieux de vaccination.
		Obstacles à la participation	RVROP_Obstacles, Participation	Freins rencontrés pour participer (temps, distance, confiance, etc.).
		Pertinence des horaires	RVRHP_Horaires, Pertinence	Pertinence des horaires proposés pour les mères de famille.
		Participation communautaire	RVRPF_Participation, Familles	Niveau de participation des parents/tuteurs aux séances.
		Difficultés rencontrées	RVRDV_Difficultés, Vaccination	Difficultés rencontrées lors des séances (temps, distance, refus du partenaire, etc.).
		Améliorations suggérées	RVRSO_Suggestions, Organisation	Recommandations pour renforcer l'organisation des campagnes.
		Suggestions d'amélioration	RVRSA_Suggestions, Accès	Suggestions pour améliorer l'accès des femmes et enfants aux séances.
**RESSOURCES**	**HUMAINES**	Disponibilité en nombre suffisant de ressources humaines	RHDSR_Disponibilité, nombre, déploiement.	Disponibilité en nombre suffisant de ressources humaines pour le déploiement (médecin, IDE, SFDE,ASC, relais communautaires, logisticiens)
		Suffisance de personnel	RHSPS_suffisance, personnel, stratégie	Suffisance de personnel pour mettre en œuvre la stratégie du rattrapage vaccinal.
		Ressources humaines couvrant l'ensemble des zones ciblées	RHSRZ_Suffisance, ressources, zones ciblées	Suffisance de ressources humaines pour couvrir l'ensemble des zones ciblées

		Existence de relais communautaires pour couvrir l'aire sanitaire.	RHERC_Existence, relais communautaire, aire sanitaire	Existence de relais communautaires pour couvrir l'aire sanitaire.
		Rôle des relais communautaires et leur niveau de disponibilité	RHRRD_Rôle, relais communautaires, isponibilité	Rôle des relais communautaires et leur niveau de disponibilité
		Relais communautaires en nombre suffisant pour couvrir l'aire sanitaire.	RHRCS_Relais communautaires, suffisant	Relais communautaires en nombre suffisant pour couvrir l'aire sanitaire.
		Disponibilité de relais communautaires pour mener les activités de vaccinations	RHDRA_Disponibilité, relais communautaires, activités, vaccinations	Disponibilité de relais communautaires pour mener les activités de vaccinations des enfants.
		Formation de relais communautaires pour mener les activités de vaccinations	RHFRA_Formation, relais communautaires, activités, vaccinations	Formation de relais communautaires pour mener les activités de vaccinations des enfants.
		Formations dispensées pour le renforcement des capacités	RHFRC_Formations, renforcement, capacités	Nombre de formations dispensées pour le renforcement des capacités
		Raison de non dispensation de formation pour le renforcement des capacités	RHRNC_Raison, non dispensation, formation, renforcement, capacités	Raison de la non dispensation de formation pour le renforcement de leurs capacités
	**LOGISTIQUE**	Disponibilité de moyens de transport en nombre suffisants.	RLDTS_Disponibilité, moyens transport, nombres suffisants.	Disponibilité de moyens de transport en nombre suffisants.
		Mobilisation suffisante de moyens de transport	RLHMT_Mobilisation, moyens, transport	Mobilisation suffisante de moyens de transport (motos, véhicules,)
		Disponibilité des outils de gestion.	RLDO_Disponibilité, outils	Disponibilité des outils de gestion.
		Disponibilité des outils de gestion	RLDOG_Disponibilité, outils, gestion	Disponibilité des outils de gestion (fiches de vaccination, carnets, registre, formulaires).
		Accessibilités aux outils	RLAON_ Accessibilités, outils nécessaires	Accessibilités aux outils nécessaires.

		nécessaires.		
		Problèmes rencontrés avec les outils, les supports de communication, ou la chaîne du froid.	RLPOF_Problèmes, outils, supports de communication, chaîne du froid.	Problèmes rencontrés avec les outils, les supports de communication, ou la chaîne du froid.
		Maintenance de la chaîne du froid.	RLMFS_Maintenance, froid, séances mobiles.	Maintenance de la chaîne du froid durant les séances mobiles.
		Difficultés en lien avec les moyens de transport, la chaîne du froid ou les intrants.	RLDFI_Difficultés, transport, chaîne du froid, intrants.	Difficultés en lien avec les moyens de transport, la chaîne du froid ou les intrants.
		résolutions mis en place pour résoudre les difficultés logistiques	RLRD_Résolutions, difficultés logistiques	Mécanismes de résolutions mis en place pour résoudre les difficultés logistiques rapidement.
		Conduite à tenir en cas de panne ou d'indisponibilité des moyens de transport	RLCPI_ Conduite à tenir, panne, d'indisponibilité.	Conduite à tenir en cas de panne ou d'indisponibilité des moyens de transport .
		Identification de solutions	RLISL_Solutions	Identification de solutions logistiques s'ils existent.
		Enumération des défis logistiques identifiés.	RLEDI_Enumération , défis, identifiés.	Enumération des principaux défis logistiques identifiés.
		Gestion des imprévus logistiques.	RLGIL_Gestion, imprévus logistiques	Gestion des imprévus logistiques.
**PERCEPTION COMMUNAUTAIRE**		Perception générale de la population sur la stratégie	PCPPS_Perception, population stratégie	Perception générale de la population sur la stratégie
		Acceptabilité de la stratégie selon les données disponibles	PCASD_Acceptabilité, stratégie, données, disponibles	Acceptabilité de la stratégie selon les données disponibles ou les remontées terrain.

		Qualification de l'acceptabilité.	PCQA_Qualification, acceptabilité.	Qualification de l'acceptabilité.
		Acceptabilité de la vaccination	PCAC_Acceptabilité, Vaccins	Niveau d'adhésion de la population à la vaccination.
		Compréhension de la stratégie	PCCP_Compréhension, Stratégie	Niveau de connaissance et de compréhension des campagnes de rattrapage.
		Acceptabilité du rattrapage	PCARV_Acceptabilité, Rattrapage	Opinion sur la pertinence et la nécessité du rattrapage vaccinal.
		Perception générale de la population	PCPGP_perception, population	Perception générale de la population sur la stratégie
		Pensé de la population relative à la stratégie de rattrapage vaccinal	PCPRV_Pensé, rattrapage vaccinal	Pensé de la population relative à la stratégie de rattrapage vaccinal.
		Raisons de la mise en œuvre de la stratégie de rattrapage vaccinal	PCRSR1_ raisons, rattrapage vaccinal	Connaissance des raisons de la mise en œuvre de la stratégie de rattrapage vaccinal.
		Compréhension de la stratégie de rattrapage vaccinal.	PCCSR_Compréhension, stratégie rattrapage, communauté	Compréhension de la stratégie de rattrapage par la communauté.
		La communication pour l'amélioration de la compréhension communautaire	PCCAC_communication, amélioration, compréhension	Contribution de la communication pour l'amélioration de la compréhension communautaire.
		Inquiétudes, rumeurs ou croyances autour de la vaccination	PCIRC_Inquiétudes, rumeurs, croyances, vaccination	Inquiétudes, rumeurs ou croyances autour de la vaccination
		Rumeurs et inquiétudes	PCRIV_Rumeurs, Vaccins	Existence de rumeurs, inquiétudes ou perceptions négatives sur les vaccins.

		Incidents de refus, des rumeurs	PCIR_ refus, rumeurs	Existence d’incidents de refus, des rumeurs.
		Refus ou de réticences	PCORR_observation, refus, réticences.	Observation de refus ou de réticences.
		Rumeurs et croyances	PCRC_Rumeurs, Croyances	Peurs, fausses informations et croyances locales autour de la vaccination.
		Existence de résistances ou rumeurs.	PCERR_Existence, résistances, rumeurs.	Existence de résistances ou rumeurs.
		Réactions face aux Inquiétudes, rumeurs ou croyances autour de la vaccination	PCRIRC_réaction, Inquiétudes, rumeurs, croyances, vaccination	Réactions face aux Inquiétudes, rumeurs ou croyances autour de la vaccination
		Observation de résistances, réticences, rumeurs ou barrières socioculturelles.	PCRBS_Résistances, réticences, rumeurs, barrières, socioculturelles.	Observation de résistances, réticences, rumeurs ou barrières socioculturelles.
		Couverture perçue	PCCPV_Couverture, Enfants	Perception de la proportion d’enfants effectivement vaccinés.
		Participation communautaire	PCPFD_Participation, Communauté	Participation des autres femmes et dynamique communautaire autour de la vaccination.
		Dialogue avec les agents de santé	PCDFA_Dialogue, Agents	Capacité et aisance des femmes à échanger avec les agents de santé.
		Renforcement de l’adhésion	PCRAC_Renforcement, Adhésion	Idées pour renforcer la confiance et l’engagement communautaire.
		Données ou retours qualitatifs disponibles sur l’acceptabilité de la stratégie.	PCDAS_Données, acceptabilité, stratégie.	Données ou retours qualitatifs disponibles (via les districts, ONG, ou feedback

				communautaire) sur l'acceptabilité de la stratégie.
		Approches pour l'amélioration de l'acceptabilité.	PCAAA_approches, acceptabilité	Approches adoptées pour l'amélioration de l'acceptabilité.
		Leçons apprises et des bonnes pratiques.	PCDLP_documentations, leçons, pratiques	Documentations des leçons apprises et des bonnes pratiques.
		Stratégies développées pour l'amélioration de la compréhension et l'adhésion des populations.	PCSCA_Stratégies, compréhension, Adhésion, populations.	Stratégies développées pour l'amélioration de la compréhension et l'adhésion des populations.
		d'opinions permettant d'améliorer la compréhension des populations rurales	PCOCR_opinions, compréhension, populations rurales	Mention d'opinions qui permettrait d'améliorer la compréhension des populations rurales à cette stratégie.
		Idées permettant d'améliorer l'adhésion des populations rurales	PCIAP_idées, adhésion	Evocation d'idées qui permettront d'améliorer l'adhésion des populations rurales à cette stratégie.
		Contribution à la vaccination les enfants par les familles	PCCVF_Contribution, familles	Contribution à faire vacciner les enfants par les familles
		Propositions apportées pour améliorer l'adhésion des familles.	PCPAF_ Propositions, adhésion des familles.	Propositions apportées pour améliorer l'adhésion des familles.
		Renforcement de la participation	PCRPI_Renforcement, Participation	Propositions pour accroître la participation et l'information des femmes.
		suggestions aux autorités sanitaires pour le renforcement de cette stratégie	PCPSS_suggestions	Proposition de suggestions aux autorités sanitaires pour renforcer cette stratégie
		Recommandation pour le renforcement de l'ancrage local	PCRRA_Recommandation, ancrage local	Recommandation pour le renforcement de l'ancrage local de ce type d'intervention.

Compréhension communautaire (non-vaccination et vaccination incomplète)		Perception de la non-vaccination	CCPNV_Perception, non Vaccination	Perception générale sur les enfants non ou partiellement vaccinés.
		Raisons de non / incomplète vaccination	CCRNVI_Raisons, Incomplète	Raisons évoquées pour la non-vaccination ou la vaccination incomplète.
		Raisons de non-vaccination	CCRNV_Raisons, non Vaccination, ZD	Raisons avancées pour expliquer la non-vaccination des enfants ZD.
		Raisons de vaccination incomplète	CCRVI_Raisons, Incomplète,	Causes de la vaccination incomplète des enfants SV (0–59 mois).
		Implication parentale	CCIPV_Implication, Parents	Conditions pour une meilleure, le niveau d'engagement et d'implication parentale dans la vaccination,
**Clôture**		Solutions proposées	CSAIP_Solutions, Implication	Mesures à mettre en place pour améliorer l'implication parentale.
		recommandations	CRSP_recommandations,suge stions	Messages finaux, recommandations ou suggestions.

LEGENDE :

COULEUR	GUIDE
BLEU	DDS DRS
NOIR	ACTEURS TECHNIQUES
ROUGE	IDE-SFDE-ASC
VIOLET	FOCUS GROUP HOMME
MARON	FOCUS GROUP FEMME

VERT	RESPONSABLE COMMUNAUTAIRE
ROSE	DCPEV ET PARTENAIRES
