# Supplementary material for: From Information to Vaccination: A Conceptual Framework for Understanding Community Participation in Catch-Up Vaccination for Children in Abidjan, Côte d’Ivoire
Source: Vaccines (Basel). 2026 Sep 8;14(9):786. doi: 10.3390/vaccines14090786 (PMC13612099; doi:10.3390/vaccines14090786)
Supplement: Supplementary file 1 [file vaccines-14-00786-s001.zip › S2. Focus Group Guide ¿C MEN in rural areas - ENG.pdf]

Focus Group Guide – MEN in rural areas

Identification of the respondent

Health region:

Health district:

Locality:

Introduction

- Introduction of the facilitator
- Introduction of the participants

  (Surname and first names, age, sex, main activity, level of education, marital status, employment status, monthly household income, urban or rural place of residence, religion/ethnic group or cultural affiliation, household size)

- Confidentiality assured
- Written consent of the participant
- Objective of the discussion: "to determine the current status of implementation of the strategy for sensitization and identification of Zero-Dose (ZD) and under-vaccinated (UV) children, to understand the difficulties related to this implementation, and to formulate recommendations to improve this strategy."

- **1. Sensitization and identification of ZD and UV children;**

Objective: Identify the types of actions carried out and their frequency

- Have you been informed about vaccination awareness campaigns in your village?
- What types of awareness-raising activities have you observed or participated in? (Public meetings, door-to-door visits, radio announcements, visits by community volunteers, etc.)
- How often have these actions taken place?
- Who conducts these actions? (health workers, traditional leaders, NGOs, etc.)
- In your opinion, are these activities effective? Why or why not?
- As men, what role do you play, or could you play, in these awareness-raising activities?

2. Vaccination catch-up / identification of ZD and UV children

Objective: Explore the organization of and participation in mobile/outreach sessions.

- Have you heard about vaccination catch-up sessions in your village or nearby areas?
- Do you know how they are organized (location, frequency, procedures)?
- Who informed you about these sessions?

- Have you accompanied your children, younger brothers/sisters, or other family members to these sessions? Why or why not?
- In your opinion, what are the obstacles or barriers to family participation?
- What should be improved in the organization of these campaigns?

3. Community perception

Objective: Understand the level of acceptability and understanding of the strategy.

- What do you think about these vaccination catch-up campaigns?
- Do you think people in your community clearly understand what they are about?
- Are vaccines well accepted here? Why?
- Are all children of vaccination age vaccinated? Why?
- Have you heard any rumors, fears, or beliefs about vaccination?
- What should be done to increase community adherence to the strategy?

4. Community understanding

Objective: Identify the reasons for non-vaccination among ZD children and/or incomplete vaccination among UV children aged 0 to 11 months and those aged 12 to 59 months.

- What do you think about incomplete vaccination among UV children aged 0 to 11 months and those aged 12 to 59 months, and about non-vaccination among ZD children? (Probe by asking why.)

- What are the reasons that lead parents not to vaccinate their children or not to complete their vaccinations?

- What would be needed for parents to become genuinely involved in vaccinating their children?

Closing

- Final remarks from the participants: suggestions or messages to be addressed to the organizers of the strategy.
- Acknowledgements.
