# Supplementary material for: From Information to Vaccination: A Conceptual Framework for Understanding Community Participation in Catch-Up Vaccination for Children in Abidjan, Côte d’Ivoire
Source: Vaccines (Basel). 2026 Sep 8;14(9):786. doi: 10.3390/vaccines14090786 (PMC13612099; doi:10.3390/vaccines14090786)
Supplement: Supplementary file 1 [file vaccines-14-00786-s001.zip › S3. Focus Group Guide ¿C WOMEN in rural areas - ENG.pdf]

Focus Group Guide – WOMEN in rural areas

Health region:

Health district:

Locality:

Introduction

- Introduction of the facilitator
- Introduction of the participants

  (Surname and given names, age, sex, main activity, level of education, marital status, employment status, monthly household income, urban or rural place of residence, religion/ethnic group or cultural affiliation, household size)

- Confidentiality assured
- Written consent of the participant

- Objective of the discussion: "to determine the current state of implementation of the awareness-raising and identification strategy for Zero Dose (ZD) and under-vaccinated (UV) children, to understand the challenges related to this implementation, and to formulate recommendations to improve this strategy."

1. Community awareness-raising

Objective: Discuss your experience with awareness-raising and vaccination campaigns

- How were you informed about vaccination activities and awareness-raising campaigns in your community?
- What forms of awareness-raising personally reached you? (women's meetings, courtyard activities, local radio stations, etc.)
- How often do these activities take place?
- Who comes to talk to you about these topics? (health workers, traditional birth attendants, neighbors, etc.)
- Do you feel well informed? If not, what is missing?
- In your opinion, how can women participate more effectively in these activities?

2. Vaccination catch-up / identification of ZD and UV children

- **Objective: Explore access to and participation in vaccination sessions**
- Have you ever participated in a mobile or outreach vaccination session?
- How did you know it was taking place?
- Where does it usually take place? Is it easy to access?
- Are the schedules convenient for mothers?
- What difficulties have you encountered (time, distance, sick children, refusal, etc.)?

- Do you have any suggestions for improving women's and children's access to these sessions?

3. Community perception

Objective: Understand the level of acceptability and understanding of the strategy.

- What do you think about vaccination catch-up? Is it a good thing? If yes, why should vaccination catch-up be carried out?
- Do other women around you participate in it?
- Are there any concerns or rumors about vaccines?
- Do you feel comfortable asking health workers questions?
- What would be needed for women to participate more and be better informed?

4. Community understanding

Objective: Identify the reasons for non-vaccination among ZD children and/or incomplete vaccination among UV children aged 0 to 11 months and those aged 12 to 59 months.

What do you think about incomplete vaccination among UV children aged 0 to 11 months and those aged 12 to 59 months, and about non-vaccination among ZD children? (Probe by asking why.)

What are the reasons that lead parents not to vaccinate their children or not to complete their vaccinations?

What would be needed for parents to become truly involved in vaccinating their children?

Closing

- Final round of comments: a message or suggestion to convey.
- Thanks and end of the session.
