# Supplementary material for: From Information to Vaccination: A Conceptual Framework for Understanding Community Participation in Catch-Up Vaccination for Children in Abidjan, Côte d’Ivoire
Source: Vaccines (Basel). 2026 Sep 8;14(9):786. doi: 10.3390/vaccines14090786 (PMC13612099; doi:10.3390/vaccines14090786)
Supplement: Supplementary file 1 [file vaccines-14-00786-s001.zip › S4. Interview Guide ¿C Community Leaders in Rural Areas- ENG.pdf]

Interview Guide – Community Leaders in Rural Areas

- **Introduction**
  - Introduction of the researcher / interviewer
  - Presentation of the purpose of the interview:

"This interview aims to better understand the role of community leaders in awareness-raising activities and the catch-up vaccination strategy in your community."

- Confidentiality assured and request for consent
- Basic information about the respondent:
  - Name or position
  - Locality
  - Years of experience in the current role

1. Community awareness-raising

Objective: Explore the roles played by leaders in providing information and mobilizing communities around health.

- Are you approached for health information or awareness-raising campaigns, particularly on vaccination?
- What types of awareness-raising activities have been organized in your locality in recent months?

(Community meetings, interventions at places of worship, group activities with young people/women, home visits, local radio, etc.)

- How often do these activities take place?
- Who initiates or coordinates these activities (health workers, NGOs, health authorities)?
- What is your role in these campaigns?
- In your opinion, which channels are most effective for reaching the local population?
- Have you received training or orientation to participate in these activities?
- What difficulties do you encounter in mobilizing the population?

2. Catch-up vaccination

Objective: Understand community involvement in organizing mobile or outreach sessions.

- Have you heard about mobile or outreach vaccination sessions in your locality?
- How are they organized? Who decides on the locations and dates?
- Are you informed in advance? Do you participate in the planning?
- What is your role in welcoming or mobilizing the population for these sessions?
- Are the sessions well attended by families? If not, why?

- What improvements do you suggest to better organize these campaigns in your community?
- Are there still children who miss vaccination? What, in your opinion, are the reasons?

3. Community perception

Objective: Identify the level of social acceptability and any potential resistance.

- In your opinion, what does the population think of the catch-up vaccination strategy?
- Is it well understood? Do people know why it is being implemented?
- Do families readily bring their children to be vaccinated?
- Have you heard concerns, rumors, or beliefs about vaccination (diseases, infertility, etc.)?
- How do you respond to such statements?
- What, in your opinion, could improve the understanding and acceptance of this strategy among rural populations?
- What suggestions would you like to convey to the health authorities to strengthen this strategy in your community?

◆ **Conclusion**

- Participant's final words: message, suggestion, or recommendation
- Thanks for the time given and the contribution
