# Supplementary material for: From Information to Vaccination: A Conceptual Framework for Understanding Community Participation in Catch-Up Vaccination for Children in Abidjan, Côte d’Ivoire
Source: Vaccines (Basel). 2026 Sep 8;14(9):786. doi: 10.3390/vaccines14090786 (PMC13612099; doi:10.3390/vaccines14090786)
Supplement: Supplementary file 1 [file vaccines-14-00786-s001.zip › S5. Interview Guide ¿C DCPEV and EPI Partners - ENG.pdf]

Interview Guide – DCPEV and EPI Partners

Introduction

- Date of interview
- Presentation of the researcher and the purpose of the study:
- Sociodemographic information (Structure/Institution/organization represented, current position held, number of years in the current position)
- Reminder of the confidential and non-nominative nature of the interview
- Request for the participant's verbal/written consent.

1. Planning and coordination

Objective: To understand the roles and responsibilities of central-level actors and partners in structuring and steering the strategy.

- Is there a national strategic plan or specific guidelines governing post-pandemic vaccination catch-up?
- What is your institution's contribution to the design or implementation of this plan?
- Is planning contextualized at the local level (districts, rural communities)?
- How is coordination organized among the different levels (national, regional, local)?
- Are populations or their representatives involved in this planning? If so, how?
- What are the main constraints encountered in multisectoral coordination?

2. Community sensitization

Objective: To identify the communication strategies used to strengthen community adherence.

- What communication strategies have been implemented at the national and local levels to support the vaccination catch-up strategy?
- What types of sensitization activities have been organized (radio broadcasts, campaigns, social mobilization, etc.)?
- How frequent are these actions?
- What tools/materials have been developed? Have they been adapted to local contexts (languages, customs, preferred channel)?
- How are community health workers and local leaders involved in these actions?
- Do you have evaluations or feedback on the impact of these sensitization campaigns?

3. Active case finding

Objective: To understand the place of active case finding in the catch-up strategy.

- Is the active case-finding strategy (door-to-door, home visits) an integral part of the catch-up plan?

- What guidelines have been issued on this matter?
- What criteria are recommended for identifying unvaccinated or under-vaccinated children?
- Have specific tools or instruments been disseminated to support this activity (lists, health cards, forms)?
- Are non-state actors (NGOs, volunteers, civil society organizations) involved in this active case finding?

4. Vaccination catch-up

Objective: To document the operational strategy for mobile or outreach sessions.

- What is the national approach for organizing mobile or outreach sessions (zoning, frequency, targeting)?
- How are localities prioritized?
- What resources (financial, human, logistical) have been mobilized to support these sessions?
- What difficulties have been encountered (community adherence, geographical accessibility, logistics)?
- Is there a monitoring and evaluation strategy for these sessions? If so, which indicators are monitored?

5. Human resources and logistics

Objective: To assess the availability of the necessary human, material and logistical resources.

- Are there sufficient human resources to cover all targeted areas?
- What is the role of community health workers and their level of availability?
- Have trainings been provided to strengthen their capacities?
- Are management tools (vaccination forms, health cards, registers, forms) available and used?
- What means of transport are mobilized (motorcycles, vehicles, NGO support)? Are they sufficient?
- Is the cold chain properly maintained during mobile sessions?
- What are the main logistical challenges identified?

6. Community perception

Objective: To analyze the level of acceptability and understanding of the strategy within communities.

- What perceptions do rural populations have of the vaccination catch-up strategy?
- Have any resistance, reluctance, rumors or sociocultural barriers been observed?

- What strategies have been developed to improve populations' understanding and adherence?
- Do you have data or qualitative feedback (through districts, NGOs, or community feedback) on the acceptability of this strategy?
- What recommendations would you make to strengthen the community dimension of this strategy?

Conclusion

Participant's final remarks

- General recommendations from the organization represented
- Thanks for the collaboration
