# Supplementary material for: From Information to Vaccination: A Conceptual Framework for Understanding Community Participation in Catch-Up Vaccination for Children in Abidjan, Côte d’Ivoire
Source: Vaccines (Basel). 2026 Sep 8;14(9):786. doi: 10.3390/vaccines14090786 (PMC13612099; doi:10.3390/vaccines14090786)
Supplement: Supplementary file 1 [file vaccines-14-00786-s001.zip › S6. Interview Guide ¿C District Manager - ENG.pdf]

Interview Guide – District Manager (Departmental or Regional Director)

Introduction

- Interview date
- Presentation of the researcher and the purpose of the study
- Sociodemographic situation (Health Region, Health District, Facility/Institution, Respondent's profession, Respondent's level of education, Current position held, Number of years in the current position)
- Confidentiality assured
- Written consent of the participant

1. **Knowledge of immunization stakeholders**

- What existing national standards, guidelines, and other documents are available to improve the strategy for awareness-raising and identification of Zero-Dose (ZD) and under-vaccinated (UV) children?
- What about the accessibility of these documents (standards and guidelines, etc.) in health facilities?
- How does your structure (regional or departmental directorate) use data collected at the level of health facilities and in the community to make decisions aimed at improving the strategy for awareness-raising and identification of Zero-Dose (ZD) and under-vaccinated (UV) children? Are there mechanisms in place (alert thresholds, etc.) that make it possible to understand shortcomings and low rates of services offered or used in order to continually implement corrective measures?

- What relationships exist between your directorate, NGOs, the community, and other structures involved in immunization in your area (other ministries, decentralized local authorities, NGOs with which the Health District collaborates and their areas of intervention; institutions and organizations with which the District collaborates), and what activities are carried out (community notables, local radio, religious leaders, etc.)?
- Is there a task-shifting policy related to vaccine demand?
- What is included in the package of activities for CHWs?

2. **Planning and coordination**

- Is there an operational plan at district level for implementing the immunization catch-up strategy?
- How is it developed? Who participates?
- How do you ensure coordination among health facilities, partners, and communities?
- What difficulties do you encounter in coordinating the strategy?

3. **Community awareness-raising**

- How does the district organize awareness-raising activities?
- Which partners or community stakeholders are mobilized?
- Have you implemented a communication strategy adapted to the local context?
- Is there an assessment of the reach of the activities carried out?

4. **Active search**

- Does the district plan an active search phase?
- What tools or methods are recommended for identifying target children?
- Have you trained staff on this component?
- How do you assess the effectiveness of this approach?

5. **Immunization catch-up**

- What is the frequency of outreach or mobile sessions?
- What criteria guide their implementation?
- What human, logistical, and financial resources are mobilized?
- How do you ensure monitoring and reporting of these activities?

6. **Human resources and logistics**

- Do you have sufficient staff to implement the strategy?
- Are management tools available (tables, cards/booklets, target lists, etc.)?
- Do you experience difficulties related to transport resources, the cold chain, or supplies?
- How do you manage logistical contingencies?

7. **Community perception**

- What is the population's general perception of the strategy?
- Do you observe refusals or reluctance?
- What approaches do you adopt to improve acceptability?
- Have you documented lessons learned and good practices?
