# Supplementary material for: From Information to Vaccination: A Conceptual Framework for Understanding Community Participation in Catch-Up Vaccination for Children in Abidjan, Côte d’Ivoire
Source: Vaccines (Basel). 2026 Sep 8;14(9):786. doi: 10.3390/vaccines14090786 (PMC13612099; doi:10.3390/vaccines14090786)
Supplement: Supplementary file 1 [file vaccines-14-00786-s001.zip › S7. Interview Guide for the technical actors of the health district - ENG.pdf]

Interview Guide

Technical actors of the health district (EPI, Monitoring and Evaluation, Communication FP)

Introduction

- Date of interview
- Presentation of the researcher and the purpose of the study
- Sociodemographic situation (Health region, Health district, Structure/Institution, Respondent's profession, Respondent's level of education, Current position held, Number of years in the current position)
- Confidentiality assured
- Written consent of the participant

1. **Knowledge of vaccination actors**

- What activities do you carry out that are related to the strategy for raising awareness and identifying Zero-Dose (ZD) and under-vaccinated (UV) children?
- Can you list the activities you carry out with health workers in your health area?
- Can you give us the reasons that lead health workers to undertake strategies for raising awareness and identifying Zero-Dose (ZD) and under-vaccinated (UV) children?

2. **Planning and coordination**

- What was your role in developing the local implementation plan?
- Is the strategy harmonized with other public health interventions?
- How is implementation monitoring ensured?
- What synergies exist between the EPI, monitoring and evaluation, and communication components?

3. **Community awareness-raising**

- What communication approaches were implemented to support the strategy?
- Did you produce or adapt specific materials?
- How were community relays involved in awareness-raising?
- In your opinion, are some channels more effective than others?

4. **Active search**
- Is active search an integrated component of the strategy?
- What tools were produced or adapted for this step?
- How is the quality of the data collected ensured?
- Have you observed any challenges or limitations in operationalization?

5. **Vaccination catch-up**
- What technical guidance did you provide for organizing the sessions?
- Did you participate in mapping or microplanning?
- How is the performance of mobile teams monitored?
- Have you noted any discrepancies between the plan and the reality on the ground?

6. **Human resources and logistics**
- Does the district have a sufficient number of resources for deployment (workers, community relays, logisticians)?
- Have you encountered problems with tools, communication materials, or the cold chain?
- What mechanisms are in place to resolve logistical difficulties quickly?

7. **Community perception**
- According to the available data or field feedback, what is the acceptability of the strategy?
- Have there been incidents of refusal or rumors?
- How has communication contributed to improving community understanding?
- What do you recommend to strengthen the local anchoring of this type of intervention?
