# Supplementary material for: From Information to Vaccination: A Conceptual Framework for Understanding Community Participation in Catch-Up Vaccination for Children in Abidjan, Côte d’Ivoire
Source: Vaccines (Basel). 2026 Sep 8;14(9):786. doi: 10.3390/vaccines14090786 (PMC13612099; doi:10.3390/vaccines14090786)
Supplement: Supplementary file 1 [file vaccines-14-00786-s001.zip › S8. Interview Guide Nurse Midwife CHW - ENG.pdf]

Interview Guide

Nurse / Midwife / Community Health Worker at the rural health center

Introduction

- Interview date
- Introduction of the researcher and the purpose of the study
- Sociodemographic information (Health region, Health district, Facility/Institution, Respondent's profession, Respondent's level of education, Current position held, Number of years in the current position, Number of years in the field of vaccination.)
- Confidentiality assured
- Written consent from the participant

Objective: To better understand the strategy for raising awareness and identifying Zero-Dose (ZD) and under-vaccinated (UV) children

1. **Knowledge of vaccination actors**

- What vaccines do you offer to children in your health area?
- Can you list the activities you carry out with community health workers in your health area?
- Can you tell us the reasons that most frequently lead the population to visit your health facility?

2. **Planning and coordination**

- Is there a local plan for implementing the awareness-raising and vaccination catch-up strategy?
- Are you involved in planning these activities? If yes, at what levels?
- Are the populations in your locality involved in this activity? If yes, how are they involved?

3. **Community awareness-raising**

- What awareness-raising actions have you implemented?
- Who is responsible for this awareness-raising activity (you, CHWs, NGOs, community leaders)?
- How often are they carried out?
- What are the preferred channels for reaching the population (door-to-door visits, radio, places of worship, etc.)?
- Are these activities well perceived by the communities? Why?

4. **Active search:**

- Do you conduct home visits to identify unvaccinated or under-vaccinated children?
- Are there criteria used to target households or children? If yes, which ones?

- Do you have tools or materials to guide this active search?
- What are the main difficulties encountered?

4. Vaccination catch-up

- How are mobile or outreach sessions organized in your health area?
- Who is responsible for implementing them?
- How frequently are outreach visits carried out?
- Do parents respond well to these sessions? If not, why? How do they organize themselves?

5. Human resources and logistics

- Do you have community relays? Are they trained and available?
- Do you have access to the necessary tools (follow-up sheet, vaccination card, consent form, etc.)?
- What means of transport are made available? Are they sufficient?
- In the event of a breakdown or unavailability, what do you do?

6. Community perception

- In your opinion, how does the community understand the catch-up strategy?
- Is there good acceptability?
- What resistance or rumors have you encountered?
- What do you suggest to improve families' adherence?
